# Supplementary material for: Optimization and Formulation of Nanostructured and Self-Assembled Caseinate Micelles for Enhanced Cytotoxic Effects of Paclitaxel on Breast Cancer Cells
Source: Pharmaceutics. 2020 Oct 18;12(10):984. doi: 10.3390/pharmaceutics12100984 (PMC7589039; doi:10.3390/pharmaceutics12100984)
Supplement: Supplementary file 1 [file pharmaceutics-12-00984-s001.pdf]

pharmaceutics

Supplementary Materials: Optimization and Formulation of Nanostructured and Self-Assembled Caseinate Micelles for Enhanced Cytotoxic Effects of Paclitaxel on Breast Cancer Cells

Farah Rehan, Nafees Ahemad, Rowshan Ara Islam, Manish Gupta, Siew Hua Gan and Ezharul Hoque Chowdhury

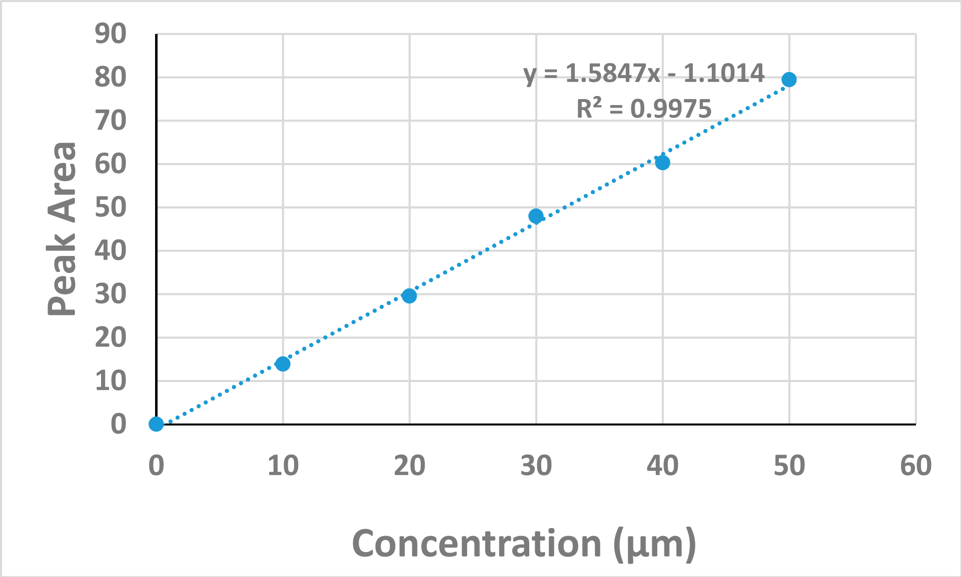

Figure S1. Standard Calibration Curve of PTX following HPLC analysis for free PTX concentration (0 uM–50 uM) of using an Agilent HPLC system with L.C. software coupled to a DAD detector and equipped with Column C18 (4.6 × 150 mm, 5 µm) maintained at 30 °C. Acetonitrile/water (55:45) was used as a mobile phase with a flow rate of 1.5 mL/min and an injection volume of 4 µL was used throughout. Detection was at 227 nm.

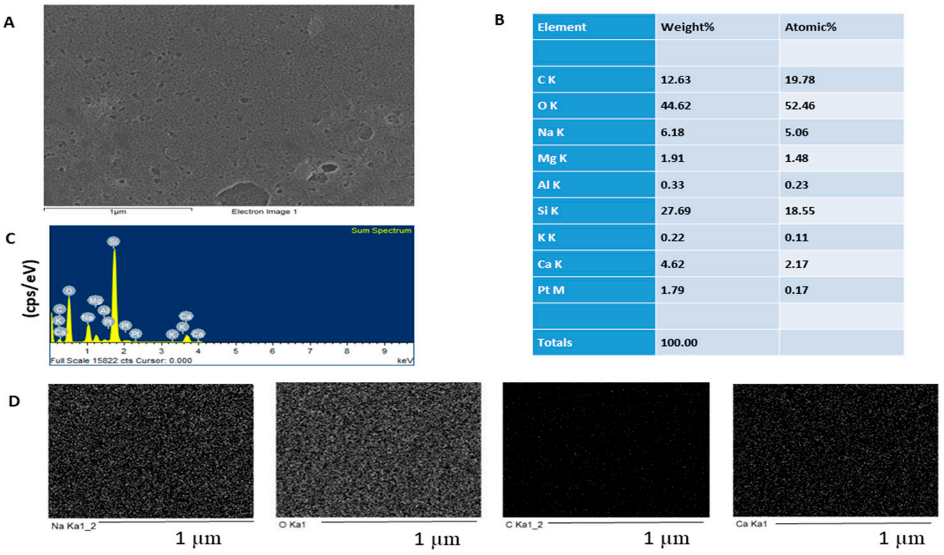

Element	Weight%	Atomic%
C K	12.63	19.78
O K	44.62	52.46
Na K	6.18	5.06
Mg K	1.91	1.48
Al K	0.33	0.23
Si K	27.69	18.55
K K	0.22	0.11
Ca K	4.62	2.17
Pt M	1.79	0.17
Totals	100.00	

Figure S2. EDX analysis (**a**) SEM image of Blank NaCNs (1 mg/mL) (**b**) sample elemental distribution in tabular form (**c**) sample elemental distribution where keV is the accelerating voltage range used for EDX analysis, and cps/eV is counts per sec per electron-volt. (**d**) SEM images showing the uniform distribution of C, O, Na and Ca elements in the sample at a scale bar of 1 µm.

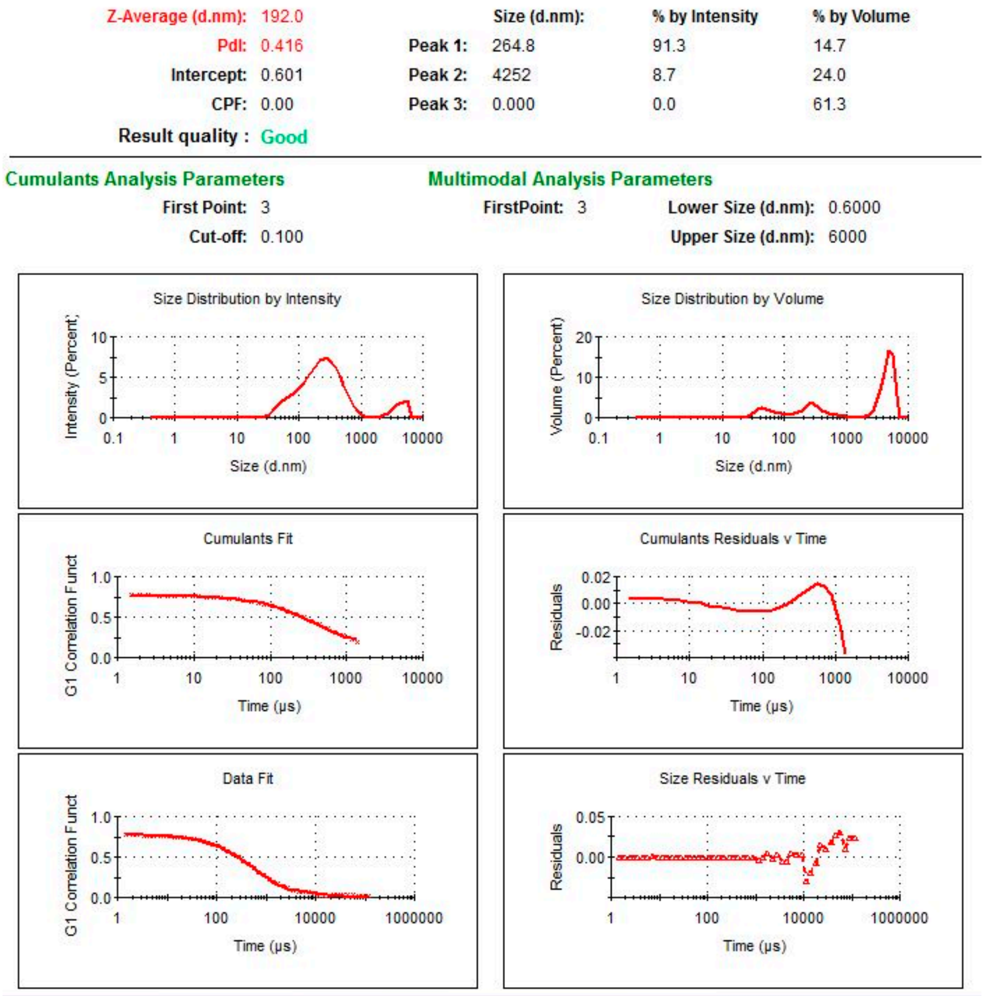

Figure S3. Graphical representation of Cumulants Analysis and the Multimodel Analysis Parameters produced via a DLS technique for determination of particle size distribution of PTX-NaCNs.
